# Exploring self-regulation theory as a mechanism of the effects of psychological contract fulfillment: The role of emotional intelligence

**DOI:** 10.3389/fpsyg.2023.1090094

**Published:** 2023-03-30

**Authors:** Lyonel Laulié, Gabriel Briceño-Jiménez, Gisselle Henríquez-Gómez

**Affiliations:** Departamento de Administración, Facultad de Economía y Negocios, Universidad de Chile, Santiago, Chile

**Keywords:** psychological contract fulfillment, emotional intelligence, self-regulation, emotional exhaustion, turnover intention, structural equation modeling

## Abstract

As self-regulation theory has increasingly been used as a theoretical lens to explain the effects of psychological contract evaluations and employee outcomes, we test whether emotional intelligence (an ability for self-regulation) is a potential moderator of these relationships. More concretely, using a multiple times survey design in an education-based organization with 247 participants, we examined whether emotional intelligence moderates the mediation effect of emotional exhaustion on the relationship between psychological contract fulfillment and turnover intentions. Using a structural equations model (SEM) framework, our results support our hypotheses that individuals with low emotional intelligence do not experience the benefits of having fulfilled psychological contracts. Psychological contract fulfillment significantly reduces the likelihood of emotional exhaustion but only for individuals with high emotional intelligence. Consequently, turnover intentions are lower for emotionally intelligent individuals who experience the fulfillment of psychological contracts. Theoretical and practical implications are discussed. We conclude our study by suggesting that emotional intelligence should be considered as a relevant individual difference in future psychological contract research.

## 1. Introduction

Decades of research on psychological contracts (PC) have shown that perceptions of positive employee-employer exchanges produce positive outcomes for organizations and individuals (Zhao et al., 2007; Rousseau, 2011). At the individual level, a central mechanism of these effects is based on self-regulation theory which posits that individuals constantly try to reach individual goals which are evaluated against real experiences. When reality does not conduce individuals to personal goals, a discrepancy is created, and an appropriate response is enacted in order to reduce the experienced discrepancy. Perceptions of discrepancies between the PC and the actual employment experience that are nonconductive to personal goals generate negative emotional responses that in turn activate reparation, renegotiation, or even exit responses (Rousseau et al., 2018). In contrast, positive perceptions of PC fulfillment activate regulatory processes that facilitate positive individual outcomes (i.e., individual behaviors, attitudes, and psychological states). A basic assumption of this theory of self-regulation and psychological contracts is the idea that individuals are indeed able to regulate themselves when high or low fulfillment is experienced. But, does this mechanism operates for all individuals? Does psychological contract fulfillment produce positive effects even on individuals with low regulatory capacities?

**Figure 1:**
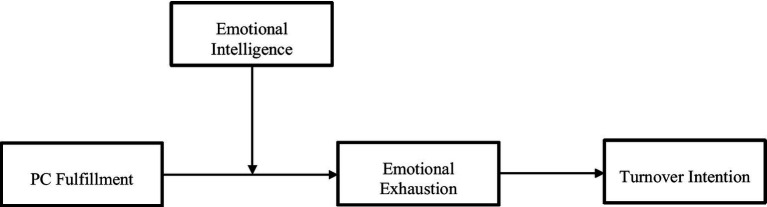
Theoretical model.

In this paper, we test the boundary conditions of self-regulation approaches to the study of psychological contracts by suggesting that some individuals may not be able to exert this regulatory mechanism. In particular, we propose that the model of self-regulation is reasonable for individuals who are able to identify, regulate, and use their emotions (i.e., emotional intelligence) but not necessarily for individuals who have not developed this ability. That is, PC fulfillment should not produce the expected positive effects on individuals with low emotional intelligence. Thus, we test the moderation effect of emotional intelligence on the relationship between PC fulfillment and individual outcomes. More specifically, our model test whether emotional intelligence moderates the mediation effect of PC fulfillment, emotional exhaustion, and turnover intention (Figure 1).

With this model, we attempt to make at least two theoretical contributions. First, we test the role of a potentially important (but so far unexplored) individual difference in the PC literature, recognizing the central role of emotion regulation in this phenomenon. Second, we test how emotional intelligence can alter the mediation of PC evaluations, emotional experiences, and behavioral intentions. Thus, we expand the boundary conditions of self-regulation perspectives in the PC literature.

In the following pages, we develop a theory that supports our model, and later we present a study of 247 individuals who responded to electronic surveys using a multiple-times design in an education-based organization. We finally discuss theoretical and managerial implications.

## 2. Theory development

### 2.1. PC theory and self-regulation

PC fulfillment is defined as the “employees’ global perceptions that, overall, the organization has fulfilled its terms of the contract in an equitable manner” (Henderson et al., 2008, p. 1,209). Social exchange theory has been the main theoretical base to explain the effects of PC fulfillment at the relational level (Blau, 1964). As individuals experience positive organizational inducements, they are motivated to continue the development of mutually beneficial relationships by reciprocating the organization (i.e., the exchange counterpart) with positive behaviors. At the individual level, however, self-regulation theory has risen recently as a more fine-tuned theory that explains the dynamics of psychological contracts (Rousseau et al., 2018). Self-regulation theory explains human behavior based on the idea of a feedback loop, where individuals monitor the achievement of personal goals, experience emotions caused by their lived experiences, and modify cognitions and behavior toward goal attainment (Lord et al., 2010).

When employment promises are fulfilled, more propitious conditions are perceived by employees, helping them reach valued personal goals, and experience positive feelings about the employment relationship and the convenience of continuing social exchanges in the future (Castanias and Helfat, 1991; Conway and Briner, 2002). The positive affect caused by PC fulfillment and perceptions of goal conduciveness have implications for the perception of exchange relationships as well (Forgas and George, 2001; Isen, 2001). Similarly, emotions play a significant role in the perceptions of fulfillment. PC fulfillment does not only create positive emotions, but it is more likely to be perceived where discrepancies do not generate negative emotions (Kiefer et al., 2022), suggesting that PC fulfillment involves both perceptions and emotions (Conway and Briner, 2002; Rousseau, 2011). Because of the important mediating role of affect in PC theory, a logical extension of these conclusions is that experiences of PC fulfillment should prevent individuals from experiencing negative emotions and exit cognitions, a result that has received meta-analytic support (e.g., Zhao et al., 2007). As Figure 1 suggests, we expect PC fulfillment to be related to turnover intention through the effects of emotional exhaustion, and we developed those relationships in the following paragraphs.

In longer periods of time, PC fulfillment should prevent individuals from having negative work experiences. For instance, previous research has shown that PC fulfillment was negatively related to emotional exhaustion (Gakovic and Tetrick, 2003; Abdalla et al., 2021), defined as the “feelings of being emotionally overextended and depleted of one’s emotional resources” (Maslach and Goldberg, 1998, p. 64). Emotional exhaustion is a phenomenon that occurs after the depletion, over time, of personal psychological resources. We expect to observe that PC fulfillment deters the occurrence of emotional exhaustion because (i) PC fulfillment injects socio-emotional resources into individuals through the effects of goal attainment and (ii) PC fulfillment reduces the need for individuals to focus on creating strategies to reduce discrepancies and obtain valued goals. To support this point, previous PC research has shown that low past PC fulfillment experiences alter present PC fulfillment (Robinson et al., 1994; Coyle-Shapiro and Kessler, 2002), buffering the effects on important outcomes such as burnout (Zhong et al., 2021).

### 2.2. The moderating role of emotional intelligence

In this article, we argue that the expected effects of self-regulation in PC theory are less likely to occur for individuals who are less capable of identifying and using the additional resources that PC fulfillment brings about. In particular, we study the moderating effect of emotional intelligence on the relationship between PC fulfillment and emotional exhaustion. Although PC research has already acknowledged the role of individual differences before (e.g., Kickul and Lester, 2001; Coyle-Shapiro and Neuman, 2004; Raja et al., 2004; Hermida and Luchman, 2013), emotional intelligence has been rarely studied as a theoretically relevant moderator.

We understand emotional intelligence (EI) as “the ability to carry out accurate reasoning about emotions and the ability to use emotions and emotional knowledge to enhance thought (Mayer et al., 2008).^1^ EI is a between-person individual difference that has been conceptualized as a multi-dimensional ability including the ability to evaluate own emotions, evaluate the emotions of others, regulate own emotions, and use emotions for goal achievement (Mayer et al., 2016). Because of these abilities, an individual’s EI has been positively associated with work performance (Joseph and Newman, 2010; O'Boyle et al., 2011) and other work criteria such as acute stress, leadership emergence, or negotiation value created (e.g., Elfenbein et al., 2007; Côté et al., 2010; Kotsou et al., 2019; Lea et al., 2019).

Moreover, EI has been a significant mechanism of self-regulation theories. It is well-established that emotional intelligence has a positive effect on emotion regulation (e.g., Laborde et al., 2014; Thomas and Zolkoski, 2020), with documented direct effects on emotional exhaustion as high IE individuals are more likely to exert higher efforts on goal striving processes, injecting positive feelings through goal achievement (e.g., Moon and Hur, 2011; Tziner et al., 2020). In addition to the direct effects of EI, individuals are also likely to interact differently with job-specific situations and contextual factors. This distinct theoretical mechanism of the effects of emotional intelligence has been dubbed the “moderation model” by EI scholars (Côté, 2014; Pradhan and Jena, 2018).

We propose that individuals with high rates of EI have a higher capacity for self-regulation that allows them to interact more positively in exchanges with the organization. First, because of their capacity to regulate their own emotions, a high EI makes individuals less vulnerable to negative disruptions, recovering faster from PC breaches. PC research suggests individuals with low self-control are more likely to be affected by PC breaches (Bal et al., 2011; Restubog et al., 2015; Balogun et al., 2018). Considering that breach is more the norm than the exception in many organizations (Robinson and Rousseau, 1994), high EI individuals are less likely to be affected by small breaches of the PC. That is, high EI may use less time in the repair phase of the PC dynamics (Rousseau et al., 2018). On the other side, the level of reactivity of negative events in individuals with low EI is high (Bechtoldt and Schneider, 2016). Lanciano et al. (2012) found that individuals that scored low EI ruminated less about previous stressful experiences.

Second, it is expected that high EI individuals can use the socio-cognitive resources injected by high PC fulfillment in helping them to better regulate their unpleasant feelings in other domains of the working experience and the consequent effect outside the workplace. In addition, high EI individuals are more aware of unpleasant feelings caused by low PC fulfillment, valuing the fulfillment of employment promises, and creating relief in emotional demands when fulfillment occurs. Previous research has shown that individuals with high EI exert increased attention to positive emotions (Szczygieł and Mikolajczak, 2017; Lea et al., 2019). In contrast, individuals with low EI have more difficulties creating strategies for self-regulation and are less likely to perceive PC fulfillment as a relevant factor that determines goal conduciveness. The burden of emphasizing negative feelings in life events and the need to constantly ruminate about strategies to reach personal goals should affect their levels of emotional exhaustion regardless of the perceptions of the fulfillment of the PC.

These arguments are consistent with a moderation effect of emotional intelligence in the negative relationship between PC fulfillment and emotional exhaustion. The PC fulfillment-emotional exhaustion relationship is expected to be observed for individuals with high EI, but not for individuals with low EI (no significant relationship; a neutralization effect). Thus, we hypothesize:

*Hypothesis 1*: Emotional intelligence moderates the negative relationship between psychological contract fulfillment and emotional exhaustion, such that the effect is stronger for individuals with high emotional intelligence.

Previous research has provided evidence for a significant relationship between emotional exhaustion and turnover cognitions and behaviors (e.g., Boles et al., 1997; Wright and Cropanzano, 1998; Karatepe and Uludag, 2007; Giao et al., 2020). Emotional exhaustion is a personal experience of energy depletion which in many cases is solved by exiting the situation that provokes that state. At the same time, the PC literature has long sustained the mediating effect of emotional experiences in the relationship between PC evaluations (breach/fulfillment) and turnover intentions. For example, in an SEM meta-analysis, Zhao et al. (2007) showed that a model with emotions as a mediator had the best fit with the data when explaining how PC evaluations impact employee outcomes, such as turnover intentions. The dynamic model of PC processes also conceptualized negative affect as an important mediator of PC evaluations and exit behaviors [See Figure 5 in Rousseau et al. (2018)]. Basically, individuals who experience negative PC evaluations are more likely to search for different employment or occupational options in order to achieve self-set goals, taking emotion as key information to create appropriate behavioral responses (Kiefer and Antoni, 2019). These relationships suggest a mediation effect where PC fulfillment predicts turnover intentions through the effects of emotional exhaustion.

This mediation effect, however, is likely to be affected by the emotional abilities of individuals. As suggested in hypothesis 1, we expect to observe high levels of emotional exhaustion for individuals with low EI in general, regardless of their perceptions of PC fulfillment. Thus, the mediation effect of PC fulfillment, emotional exhaustion, and turnover intentions is expected to occur for individuals high in EI, but not necessarily for individuals low in EI.

*Hypothesis 2*: The mediating role of emotional exhaustion in the relationship between psychological contract fulfillment and turnover intention is moderated by employee emotional intelligence, with the effect being more substantial for individuals with high emotional intelligence.

## 3. Materials and methods

### 3.1. Data collection

To test our hypotheses, we collected quantitative data as part of a larger study about psychological contracts in teams. Participants were recruited from 8 educational institutions and one administrative department of a well-known municipality in Chile. We received support to conduct this study from the municipality as part of a series of organizational initiatives to improve HR management practices. The data was obtained using two-wave self-administered web-based surveys, which were separated by a lag time of 2 months between each survey. This was intended to reduce the potential of common method variance (Podsakoff et al., 2003). In the first survey, we collected PC fulfillment and emotional intelligence, whereas, in the second survey, we collected data about emotional exhaustion and turnover intentions. The data was matched by the research team using employee IDs, following recommendations of our Institutional Ethics Committee to protect participants’ confidentiality.

The HR department provided the updated personnel records of the organization, including emails from 247 employees. Both surveys were distributed to their email addresses through Qualtrics’ mailing tool. The sample size was 181 responses for the first survey and 176 responses for the follow-up survey. In sum, the sample consisted of 150 matched responses, including employees who answered both surveys. The final sample only included subjects who successfully completed the two surveys (137 individuals) and who successfully answered an attention check item (we eliminated 23 responses due to lack of attention). This decision improved the quality of the collected data, providing a final sample of 114 responses. Our final sample size was lower than we expected, although high enough to make a rigorous test of our main hypotheses. All employees were between 25 and 64 years old (Mean = 39.16 years, S.D. = 9.89). Most participants were female (82%) and the tenure ranged from 0.16 to 35 years (Mean = 7.55 years, S.D. = 8.00).

### 3.2. Measures

All measures of this study were applied using 5-point Likert scales (1: Completely disagree – 5: Completely agree). The surveys were applied in their Spanish versions, which can all be found in escalas.unegocios.cl. *Psychological contract fulfillment* was captured using Robinson and Morrison’s (2000) classic scale of global PC fulfillment. To better align our theory to our measure, we only used the fulfillment items, eliminating two negatively worded items, addressing previous concerns that Robinson and Morrison’s measure capture both fulfillment and breach (Conway and Pekcan, 2019). This procedure was already utilized in Laulié (2017) in a similar population, showing excellent psychometric properties. A sample item is “Almost all of the promises made to me by my employer have been kept so far.” Cronbach’s alpha was 0.93. To capture *emotional intelligence*, we used an ability-based EI instrument developed by Law et al. (2004) and validated in Spanish by Madrid (2020). The instrument contemplates four dimensions, including recognition of own emotions, recognition of emotions in others, regulation of own emotions, and use of emotions. An item example is “I have good control of my emotions.” Cronbach’s alpha was 0.87. *Emotional exhaustion* was captured using an 8-item subscale of the Oldenburg Burnout Inventory and validated by Halbesleben and Demerouti (2005). A previous Spanish version of this scale was used by Laulié et al. (2022). An item example is “During my work, I often feel emotionally drained.” Cronbach’s alpha was 0.86. Finally, *turnover intention* was captured by participants using a 3-item scale developed by Cammann et al. (1983). A sample item is “I often seriously think about quitting.” Alpha was 0.85. All descriptive statistics are included in Table 1.

**Table 1:** Descriptive statistics and correlations.

Variable	Mean	SD	Min	Max	1	2	3	4
1. Psychological contract fulfillment	4.14	0.84	1.00	5.00	(0.93)			
2. Emotional intelligence	4.23	0.44	3.25	5.00	0.13	(0.87)		
3. Emotional exhaustion	2.77	0.68	1.13	4.50	−0.15	−0.40**	(0.86)	
4. Turnover intention	1.90	0.99	1.00	5.00	−0.41**	−0.11	0.15	(0.85)

The table shows means, standard deviations, minimums, maximums, and intercorrelations of Individual Psychological Contract Fulfillment, Emotional Intelligence, Emotional Exhaustion, and Turnover Intention. **denotes significancy at 0.01 level (2-tailed). Alpha scores on parentheses.

### 3.3. Analyses

First, we used confirmatory factor analysis (CFA) to evaluate our measurement model. To test model fit, we used typical indexes and some of the commonly used standards in the literature (CFI > 0.90, RMSEA<0.08, SRMR<0.08). Our theoretical model was then compared against alternative measurement models. Second, to test our main hypotheses, we used structural equation modeling (SEM). This approach can reduce bias in the calculation of estimates in a mediation or moderation analysis by managing the effects of measurement error (see Ledgerwood and Shrout, 2011). For hypothesis 1, we used a full indicator method for latent interaction constructs (Marsh et al., 2013; Collier, 2020). For this method, we created interaction terms for each pair of mean-centred indicators of the independent and moderator variables (PC fulfillment and emotional intelligence, respectively), which then serve as indicators of a latent interaction unobserved construct. Third, to test hypothesis 2, we calculated an index of moderated mediation (Hayes, 2015; Hayes et al., 2017) and observed the indirect effects of PC fulfillment on turnover intentions through the effects of emotional intelligence depending on the level of the moderator variable (emotional intelligence). All analyses were carried out using SPSS, AMOS, and the PROCESS macro.

## 4. Results

### 4.1. Confirmatory factor analysis

We first examined the measurement model that form our key constructs (PC fulfillment, emotional intelligence, emotional exhaustion, and turnover intention). The hypothesized model (see Table 2) indicates an overall excellent fit of a four-factor model (χ^2^ = 111.402, df = 107, CFI = 0.99; RMSEA = 0.019; SRMR = 0.076). Then, we tested several alternative models, merging some latent constructs (models 2 to 6 in Table 2). All the alternatives resulted in a worse fit to the data. These results suggest that individuals differentiate the four constructs, providing evidence for discriminant validity.

**Table 2:** Confirmatory factor analysis.

Model	*χ* ^2^	df	*p*-value	CFI	RMSEA	SRMR	AIC	BIC
Hypothesized 4-factor model	111.402	107	0.366	0.995	0.019	0.076	203.402	329.267
3-factor model (EI and PCF are merged)	221.929	110	0.000	0.868	0.095	0.142	307.929	425.585
3-factor model (EI and EE are merged)	191.549	110	0.000	0.904	0.081	0.098	277.549	395.206
3-factor model (EE and PCF are merged)	367.957	110	0.000	0.697	0.144	0.195	453.957	571.614
2-factor model (PCF and EI are merged, and EE and TI are merged)	332.299	112	0.000	0.741	0.132	0.165	414.299	526.484
Single-factor model	542.058	113	0.000	0.496	0.183	0.216	622.058	731.506

*N* = 114; variables included: EI, Emotional Intelligence; PCF, Psychological Contract Fulfillment; EE, Emotional Exhaustion; TI, Turnover Intention; CFI, Comparative Fit Index; RMSEA, Root-Mean-Square-Error of Approximation; SRMR, Standardized Root-Mean-Square Residual; AIC, Aikake’s Information Criterior; BIC, Bayesian Information Criterion.

### 4.2. Hypothesis testing

We also created a structural equation model to evaluate our hypothesized relationships. These results are summarized in Table 3. The initial model containing only the predictor, mediator, and outcome shows an excellent fit to the data (χ^2^ = 50.282, df = 56, CFI = 1.00; RMSEA = 0.00; SRMR = 0.055) and provides evidence to confirm some of the expected relationships. PC fulfillment (*β* = −0.20, *p* < 0.10) was significantly related to emotional exhaustion, with higher fulfillment reducing the levels of emotional exhaustion. At the same time, emotional exhaustion was significantly related to turnover intentions (*β* = 0.28, *p* < 0.01). These results are aligned with PC theory and previous findings in the literature.

**Table 3:** SEM hypothesis testing.

Relationships	Unstandardized estimates	*p*-values
PCF → EE	−0.20	0.075
PCF → TI	−0.49	0.000
EE → TI	0.28	0.008
Model fit		
*χ* ^2^	50.282	
df	56	
CFI	1.00	
RMSEA	0.000	
SRMR	0.055	
AIC	120.282	
BIC	216.049	
Moderation test		
EI → EE	−0.88	0.000
H1: PCF x EI → EE	−0.81	0.013

*N* = 114; variables included: EI, Emotional Intelligence; PCF, Psychological Contract Fulfillment; EE, Emotional Exhaustion; TI, Turnover Intention; CFI, Comparative Fit Index; RMSEA, Root-Mean-Square-Error of Approximation; SRMR, Standardized Root-Mean-Square Residual; AIC, Aikake’s Information Criterion; BIC, Bayesian Information Criterion.**denotes significancy at 0.01 level (2-tailed).

**Figure 2:**
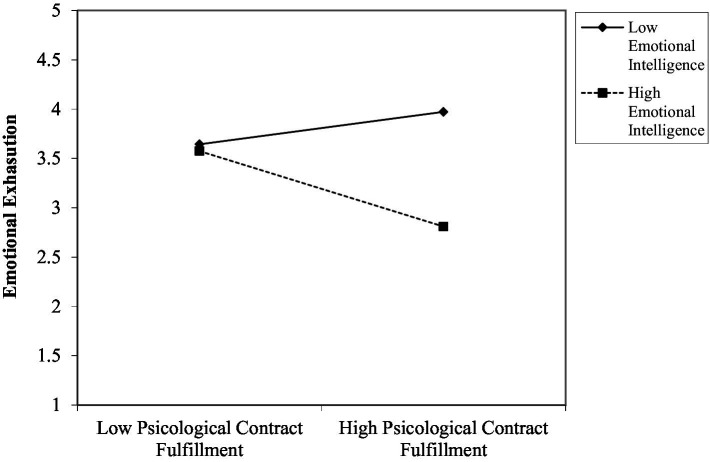
Moderation effect of emotional intelligence on PC fulfillment and emotional exhaustion.

Hypothesis 1 predicted a moderation effect of emotional intelligence on the relationship between PC fulfillment and emotional exhaustion. Our results show that, after including emotional intelligence as a predictor of emotional exhaustion (*β* = −0.88, *p* < 0.01), the latent interaction construct was significantly related to emotional exhaustion (*β* = −0.81, *p* < 0.05), providing evidence to support our hypothesis. Figure 2 plots the shape of the moderation effect. As hypothesized, when emotional intelligence is high, there is a negative relationship between PC fulfillment and emotional exhaustion. In contrast, when emotional intelligence is low, the relationship is neutralized (not significant). Individuals with low emotional intelligence presented on average high levels of emotional exhaustion. This evidence supports hypothesis 1.^2^,^3^

Hypothesis 2 predicted a moderated mediation effect of emotional intelligence on the mediation of PC fulfillment, emotional exhaustion, and turnover intention. Table 4 shows the main results of this test. When emotional intelligence is low or average, the indirect effects were not significantly different from zero. In contrast, when emotional intelligence was high, the indirect effect was significant. The index of moderated mediation was also significantly different from zero, providing evidence to support hypothesis 2.

**Table 4:** Test of moderated mediation.

Values of the moderator (EI)	Indirect effect estimate	LLCI 95%	ULCI 95%
−1 SD EI	0.059	−0.017	0.611
Mean EI	−0.039	−0.148	0.007
+1 SD EI	−0.137	−0.834	−0.022
	Estimate	LLCI 95%	ULCI 95%
Index of moderated mediation	−0.221*	−1.940	−0.027

The indirect effect is moderated by Emotional intelligence (EI). Unstandardized coefficients reported. Bootstrap sample = 5,000 with replacement.

## 5. Discussion

In this article, we proposed that emotional intelligence is a relevant individual difference that can explain the mediation of PC fulfillment, emotional exhaustion, and turnover intention. Our results support that proposition, posing interesting implications for future research.

Considering emotional intelligence as an ability that has been related to self-regulation capacities, our study provides a reasonable way to empirically test the explanatory power that self-regulation theory has on explaining how psychological contracts operate in individuals. Our results suggest that PC fulfillment is directly related to outcomes such as emotional exhaustion and turnover intentions, but only for individuals with high regulatory capacities. That is, the socio-emotional resources that PC fulfillment injects may not be sufficient for individuals with low emotional intelligence, who display higher levels of emotional exhaustion regardless of their perceptions of PC fulfillment. Thus, our results provide interesting boundary conditions of Rousseau et al. (2018) theory of PC and self-regulation.

From the point of view of social exchange theory, our results also suggest that social exchanges may be more productive for individuals with high emotional intelligence. Although decades of research have shown that creating trust in social exchanges is a ubiquitous phenomenon in social psychology (e.g., Colquitt et al., 2007), our results show that there may be individuals who are more apt to establish more beneficial social exchanges with other parties than others. These individuals are more likely to perceive organizational inducements as positive resources that can benefit their welfare. In contrast, individuals with high emotional intelligence are also more likely to exit poor exchanges. Our test of moderated mediation suggests that only individuals with high emotional intelligence are more likely to have higher turnover intentions when experiencing low PC fulfillment. That is, these individuals are more likely to exit relationships that are not fulfilling or beneficial for them. In addition, future research should explore whether other variables that could be markers of self-regulation abilities (different than emotional intelligence) may create similar boundary conditions of the effects of PC fulfillment on relevant employee outcomes.

A puzzling possibility that our findings suggest, is that as emotionally intelligent individuals are more likely to identify beneficial or detrimental relationships with the organization, they may also affect the perception of other individuals in the same group of workers (e.g., Lopes et al., 2004; Jamshed and Majeed, 2019). Based on theories of social influence, PC theory has suggested in the past that PC evaluations can be shared by specific individuals (Ho, 2005; Ho and Levesque, 2005) or teams as a whole (Laulié and Tekleab, 2016; Tekleab et al., 2020). Future research should explore whether emotionally intelligent individuals are more likely to influence other individuals’ perceptions of the PC (Griep et al., 2019).

Our findings have additional implications for the PC literature. For instance, a plausible explanation of some of the weak effects of PC fulfillment on outcomes may be the result of having samples with individuals with lower emotional intelligence. For example, Zhao et al. (2007) found a small relationship between PC breach and individual performance. We speculate that that relationship should be stronger for highly emotionally intelligent individuals. We recommend future research to use emotional intelligence as a relevant control for future PC research.

Our results also show interesting and unexpected results. Although our main goal was to contrast individuals with high vs. low emotional intelligence, the results show that the mediation of PC fulfillment, emotional exhaustion, and turnover intention was not significant for average emotional intelligence. Future research should investigate whether this result was due to a lack of power or some idiosyncratic characteristic of our sample, or whether it is a more generalizable property of the effects of PC evaluations. Similarly, future research should replicate these results in samples from other countries or industries in order to study its external validity.

We would like to finish this article by acknowledging some strengths and limitations. First, our final sample size was lower than we expected, although high enough to make a rigorous test of our main hypotheses. Second, we were not able to control for job characteristics or other individual differences that may interact with our key constructs. Future research may expand this possibility. Third, our sample and methodology have several strengths worth mentioning, such as the use of attention checks to improve the quality of our data, and the use of multiple surveys to reduce the potential problem of common method bias. We believe this study makes several contributions to the PC literature.

## 6. Conclusion

Representing the employment “deal in the mind,” PCs have been theorized to have powerful effects on employee outcomes through self-regulation processes that are activated when high or low fulfillment is experienced. In this paper, we explored whether this theoretical mechanism operates for all individuals. Our results show that the answer is “no.” Individuals with low emotional intelligence seem to be unaffected by positive or negative evaluations of the PC. We hope our results expand the PC literature and encourage future research to better understand how individual differences change the experience of the employment relationship.

## Data availability statement

The datasets presented in this study can be found in online repositories. The names of the repository/repositories and accession number(s) can be found at: https://osf.io/csdk8/?view_only=f690e5bc4a454573a1672ef30f857e01.

## Ethics statement

The studies involving human participants were reviewed and approved by Comité de Ética de la Facultad de Economía y Negocios de la Universidad de Chile. The patients/participants provided their written informed consent to participate in this study.

## Author contributions

Using CRedIT Taxonomy, the authors declare the following contributions: LL and GH-G: conceptualization. GB-J: data curation and software. GB-J, LL, and GH-G: formal analysis and investigation. LL: methodology, project administration, resources, supervision, and original draft. LL and GB-J: validation and visualization. All authors contributed to the article and approved the submitted version.

## Funding

LL would like to thank the support of the Agencia Nacional de Investigación y Desarrollo de Chile, ANID FONDECYT/INI 11190044.

## Conflict of interest

The authors declare that the research was conducted in the absence of any commercial or financial relationships that could be construed as a potential conflict of interest.

## Publisher’s note

All claims expressed in this article are solely those of the authors and do not necessarily represent those of their affiliated organizations, or those of the publisher, the editors and the reviewers. Any product that may be evaluated in this article, or claim that may be made by its manufacturer, is not guaranteed or endorsed by the publisher.

## Footnotes

^1^We take this definition in contrast to other trait-based models (Petrides and Furnham, 2003; Petrides, 2011).

^2^As a robustness check, we tested this model with and without other controls (age, tenure, gender), but the results were not altered by the inclusion of controls (these results are also available upon request to the corresponding author).

^3^An alternative explanation would be that individuals with low emotional intelligence tend to see personal situations as more negative, and thus this effect may create a negative bias in perceptions of PC fulfillment. This possibility seems less plausible as the correlation of PC fulfillment and emotional intelligence is low and not significant (Table 1).
